# Utility of platforms Viteks MS and Microflex LT for the identification of complex clinical isolates that require molecular methods for their taxonomic classification

**DOI:** 10.1371/journal.pone.0218077

**Published:** 2019-07-03

**Authors:** María Florencia Rocca, Rubén Barrios, Jonathan Zintgraff, Claudia Martínez, Lucía Irazu, Carlos Vay, Mónica Prieto

**Affiliations:** 1 Laboratorio Bacteriología Especial, Departamento de Bacteriología, Instituto Nacional de Enfermedades Infecciosas (INEI)–Administración Nacional de Laboratorios e Institutos de Salud (ANLIS) “Dr. Carlos G. Malbrán”, Ciudad Autónoma de Buenos Aires, Argentina; 2 Laboratorio de Bacteriología, Hospital Italiano de Buenos Aires, Ciudad Autónoma de Buenos Aires, Argentina; 3 Laboratorio Bacteriología Clínica, Departamento de Bacteriología, Instituto Nacional de Enfermedades Infecciosas (INEI)–Administración Nacional de Laboratorios e Institutos de Salud (ANLIS) “Dr. Carlos G. Malbrán”, Ciudad Autónoma de Buenos Aires, Argentina; 4 Instituto Nacional de Enfermedades Infecciosas (INEI)–Administración Nacional de Laboratorios e Institutos de Salud (ANLIS) “Dr. Carlos G. Malbrán”, Ciudad Autónoma de Buenos Aires, Argentina; 5 Instituto de Fisiopatología y Bioquímica Clínica, Hospital de Clínicas José de San Martín, Facultad de Farmacia y Bioquímica, Ciudad Autónoma de Buenos Aires, Argentina; Fisheries and Oceans Canada, CANADA

## Abstract

Mass spectrometry has revolutionized the clinical microbiology field in America’s and Europe’s industrialized countries, for being a fast, reliable and inexpensive technique. Our study is based on the comparison of the performance of two commercial platforms, Microflex LT (Bruker Daltonics, Bremen, Germany) and Vitek MS (bioMérieux, Marcy l´Etoile, France) for the identification of unusual and hard-to-diagnose microorganisms in a Reference Laboratory in Argentina. During a four-month period (February–May 2018) the diagnostic efficiency and the concordance between both systems were assessed, and the results were compared with the polyphasic taxonomic identification of all isolates. The study **included 265 isolates: 77 Gram-Negative Bacilli, 33 Gram-Positive Cocci, 40 Anaerobes, 35 Actinomycetales, 19 Fastidious Microorganisms and 61 Gram-Positive Bacilli.** All procedures were practiced according to the manufacturer’s recommendations in each case by duplicate, and strictly in parallel. Other relevant factors, such as the utility of the recommended extraction protocols, reagent stability and connectivity were also evaluated. Both systems correctly identified the majority of the isolates to species and complex level **(82%, 217/265)**. Vitex MS achieved a higher number of correct species-level identifications between the gram-positive microorganisms; however, it presented greater difficulty in the identification of non-fermenting bacilli and a higher number of incorrect identifications when the profile of the microorganism was not represented in the commercial database. Both platforms showed an excellent performance on the identification of anaerobic bacteria and fastidious species. Both systems enabled the fast and reliable identification of most of the tested isolates and were shown to be very practical for the user.

## Introduction

Traditionally, the bacterial and fungal identification from clinical samples has been based on the observation and analysis of microscopic, cultural, and metabolic characteristics through the use of special stains, conventional biochemical tests **[1]**, miniaturized galleries and automated methods. These are laborious procedures that can require a long time to reach a definite result. This has a direct impact on the therapeutic and epidemiological aspects, for it is known that the earlier and more appropriate the antimicrobial treatment is, the lower the patients mortality will be, especially for the ones that are in a serious condition. Nowadays, the gold-standard method for the bacteria that cannot be identified by traditional techniques is the genomic analysis. However, this methodology can be costly and tends to be difficult to implement in a conventional microbiology laboratory **[2–4]**.

MALDITOF MS mass spectrometry (*matrix-assisted laser desorption/ionization time-of-flight mass spectrometer*), has revolutionized the diagnostic in the clinical microbiology field **[5, 6]**; its basic principle consists in the generation of a characteristic mass spectrum that allows the fast and reliable identification of clinically-relevant microorganisms **[7]**.

Its use dates back to the late 80’s, when it was observed that an ultraviolet laser incidence and the sealing of the sample with an organic matrix, a soft ionization was obtained and it gave rise to the adequate fragmentation for the detection of labile molecules such as proteins, peptides, sugars and oligonucleotides **[8]**.

Later on, it begun to be applied in proteomics for the identification of microorganisms through the peptide fingerprinting method, based on ribosomal proteins since it maintains a highly conserved structure over the time **[9]**.

However, it wasn’t until 2008 when, simultaneously, Mellmann et al. in Germany **[10]** and Degand et al. in France **[11]** reported the performance of MALDI TOF for the identification of a great number of non-fermenting gram-negative bacilli obtained from clinical samples. From that moment on, there has been an exponential increase in the amount of publications and reports on the use of this technology in clinical microbiology. Its application has been subjected to multiple challenges in the past few years; such as performance evaluation in diverse taxonomic groups **[12–19]**, detection of antimicrobial resistance **[20]** and epidemiologic and subtyping tests **[21, 22].**

At the moment, there are two mass-spectrometry identifications systems available in the market: Microflex LT (Bruker Daltonics, Bremen, Germany) y Vitek MS (bioMérieux, Marcy l´Etoile, France), both have been approved by the Food and Drug Administration, USA (FDA, regulation 510k) during the year 2013, for their use in clinical microbiology diagnosis in humans. However, this validation has included only some of the species of the taxonomic groups that are clinically more relevant **[23, 24]**.

Within the framework of the National System of Laboratory Networks in Argentina, a microbiological identification network by proteomic technology (RENAEM) was created, with the spirit of promoting integration and establishing a link between the users of new technologies in the country.

The aim of this work is to evaluate the performance of both commercial platforms in the identification of a great variety of hard-to-diagnose microorganisms that are derived to Argentina’s National Reference Laboratory for their complete characterization and the transference of the evidence to the participants of the RENAEM.

Among the tested taxonomic groups, the *Nocardia* species constitute the aerobic Actinomycetales most-frequently isolated in human infections, especially in immunosuppressed patients, and their identification by MALDI TOF MS continues to be a challenge **[25]**, which is why we evaluate several extraction protocols recommended to improve the quality of the obtained spectrum; however, we consider strictly necessary the incorporation of reference profiles of fully characterized Actinomycetales species in order to improve the performance of the current commercial Databases **[26].**

## Materials and methods

The study was conducted between February and May 2018 in the Special Bacteriology Laboratory within the National Reference Institute INEI-ANLIS “Dr Carlos G. Malbrán”. Buenos Aires, Argentina.

### Strains and isolates

A total of 265 isolates were selected, representing 77 genera and 143 clinically relevant species. 77 Gram-Negative Bacilli were included (GNB), 40 Anaerobic bacteria, 19 Fastidious microorganisms (HACEK), 16 Gram-Positive Aerobic Bacilli, 45 Coryneiform microorganisms, 33 Gram-Positive Cocci and 35 aerobic Actinomycetales.

Out of the total number of isolates that were used in the study, 226 (85%) came from clinical samples, such as blood (70), sputum (53), sterile liquids (18), skin and soft tissue infection (60), other non-sterile sites (22), unknown origin (3), that were transferred to the Reference Laboratory for their complete identification. The panel was supplemented with 39 strains from the Special Bacteriology Laboratory’s Culture Collection, belonging to the Culture Collections Federation for Latin America (FELACC) in order to achieve a representative sample in terms of variability, this information can be found in Supplementary S1 Table (S1 Table).

The microorganisms were cultured in the appropriate growth media accordingly; Trypticase Soy Agar, Chocolate Agar and Columbia Agar with 5% Sheep Blood, then incubated for 24–48 hours at 37°C, in aerobic, microaerophilic or anaerobic conditions; and the results obtained by MALDITOF MS were compared with conventional biochemical tests, microscopic and macroscopic observation of colonies and miniaturized galleries API (bioMérieux, Marcy l'Étoile, France), enzymatic and serotyping tests in special cases.

The molecular identification of all the isolates included in this study was performed through the gold-standard methods according to CLSI standards **[27]**; sequencing and amplification of 16S *ARN*r gene was carried out using the primers corresponding to the position 8-27F (5′-AGAGTTTGATYMTGGCTCAG-3′) and 1492R (5′-ACCTTGTTACGACTT-3′) of the 16S *ARN*r gene of *Escherichia coli* as it has been described. **[27,28]**. In the case of complex taxonomic groups, the confirmation of the identification was done using specific primers; *rec*A gene for the isolates belonging to the *Burkholderia cepacia* complex **[29];** *rpo*B gene for Corynebacterium species **[30]** and *sec*A gene on the Actinomycetales isolates **[31].**

PCR products were sequenced using the Big Dye Terminator v3.1 Cycle sequencing kit equipment (Applied Biosystems) and analyzed in the ABI 377 Genetic Analyzer (PE Applied Biosystems).

The sequences obtained were compared with standard sequences deposited in the NCBI Gene Bank (National Center for Biotechnology Information; http://www.ncbi.nlm.nih.gov/genbank), using the BLAST V 2.0 software (Blast Internet Services, Pittsboro, NC, USA) and interpreted according to CLSI standards **[27, 28].**

### MALDI TOF mass spectrometry analysis

The assay was carried out strictly in parallel, in a similar manner as the one described by Lévesque et al. **[18]**. Each isolate was processed on the same day by a single operator and tested by duplicate in both platforms. The matrix used was the one recommended by the manufacturers; HCCA (saturated solution of α-hydroxy 4-cyanocinnamic acid dissolved in 50% acetonitrile and 2.5% trifluoroacetic acid).

### Microflex LT

Microflex LT mass spectrometer was used in a positive linear mode (laser frequency N2: 60 Hz, ion source voltage I: 20 KV, ion source voltage II: 16,7 KV, mass range of the detector: 2000–20000 Da) and the FlexControl and Biotyper RTC softwares for the entry, reading of samples and analysis of results.

As per manufacturer’s recommendations, a wooden stick was used to pick up a small portion of an isolated colony from the microorganism in an exponential growing phase and then deposited on two wells of the steel plate (Bruker Daltonics). After drying, it was covered with 1ul HCCA (Sigma-Aldrich). It was also opted to add a drop of 70% formic acid (Sigma-Aldrich) prior to the sealing with the matrix in cases where reliable identification was not obtained by the direct method.

Aerobic Actinomycetales continue to be a challenge, since they do not produce adequate mass spectrum for the identification in MALDI-TOF MS if they are processed by the direct method. This issue is related to a low number of reference bacterial spectrum in Databases (DB) and to the complex chemical composition of the cell wall of these microorganisms. With the objective of overcoming these limitations, three extraction protocols recommended for Microflex LT were tested; the ethanol and formic acid method recommended by the manufacturer (EFAE, Bruker), the NECLC method recommending the use of beads, Segawa et al. **[32]** and the HTEM (Bruker). Both the NECLC method and the HTEM were procedures difficult to carry out and they did not show peaks in most cases. Based on the results, it was opted for the EFAE Bruker method, which allowed to obtain reproducible spectrum in all cases, and showed correct identifications even at the species level. Therefore, for the Actinomycetales group we conducted a first plating through the direct method, and if a reliable identification was not achieved, we tested the EFAE Bruker extraction method as detailed: 3–4 colonies of the microorganism were inoculated in 300 ul Ultrapure Water and vortex was vigorously applied during 5–10 minutes. Then, 900 ul of pure ethanol were added and they were centrifuged at 20000g for 3 minutes. The supernatant was discarded and 20 ul of 70% formic acid and 50 ul of acetonitrile were added; vortex 2–3 minutes and centrifuged at 20000g during 3 minutes. Finally, 1 ul of the supernatant was seeded in the well of a plate and covered with 1 ul HCCA.

Once the samples with the matrix were dried, the plates were inserted in the previously calibrated equipment with the bacterial standard BTS (Bruker Daltonics) provided by the manufacturer.

The obtained spectrum was analyzed and compared with the reference spectra from the commercial DB Bruker Biotyper 3.1 (7311 MSPs or reference profiles). According to the following criteria; score values ≥ 2.0 were considered as reliable identification at the species level, while values between 1.70–1.99, indicated correct identification at the genus level, and score values <1.70 were considered non-identification. In addition, the 10% divergence criteria **[11, 33–35]** were applied between the first and the following different one of the top ten, in the case that several species showed a high score value.

If a 10% difference could not be achieved among the obtained score values, it was considered identification at the genus level (if the first and second species belonged to the same genus) or non identification (NI; if they were from different genera).

All the tests were carried out in duplicate, therefore, the plate with the highest score value was considered the final result.

### Vitek MS

Most of the methodology is similar in both platforms. A small portion of the microorganism was placed on the disposable plate (bioMerieux, Ref.410893) provided by the manufacturer. Each stain was covered with 1ul of matrix (Vitek).

For the aerobic Actinomycetales, the efficiency of the extraction kit VITEK MS MYCOBACTERIUM/NOCARDIA KIT (bioMerieux, Ref. 415659) was evaluated over 24 isolates belonging to the genus *Nocardia* sp., *Rhodococcus* sp., *Gordonia* sp., *Tsukamurella* sp.

In the same way, and with the purpose of improving the identification in Vitek MS for this complex group of microorganisms; we tested the same isolates, but using the following ***in-house* protocol**: the sample was seeded in a well through the direct method by duplicate; 1ul of Formic Acid (Vitek, Ref.411072) was added and pipetted several times in order to achieve the disruption in situ; then approximately 0.5 ul were transferred to the second well and sealed with 1 ul of HCCA.

The standard strain *Escherichia coli* ATCC 8739 was used as a calibrator and internal quality control for each acquisition group, manipulated under strict conditions (isolated in Columbia Agar with 5% Sheep Blood and incubation for 18–24 hours at 37°C).

The plate was placed in the instrument and once the vacuum level was reached, the sample processing and reading started. The obtained spectra characteristics were compared with the ones from the Recognition Database IVD KB 3.0 for in-vitro diagnosis (1046 species) since it is the simplest one and it is used for routine identifications in the clinical laboratory. The results were validated through the MYLA software. This software calculates confidence levels; values between 60.0 and 99.9 indicate reliable discrimination at a species / group level. Low-discrimination identifications occur when there is more than one significant organism / group, but no more than 4. In this case, the sum of the confidence values is equal to 100.

When there is similarity with more than 4 organisms or a coincidence is not found, it is considered as Non-Identification (NI).

If a satisfactory result was not obtained, the change to the RUO DB (1857 taxa) was selected, this database is more complex to use and basically intended for research. The software for analysis of results in this DB, is the Saramis Premium V4.15.0.

### Statistical qualitative analysis

To compare the performance of the two platforms, a concordance analysis was made between the results. Agreement of positivity result was assessed with kappa statistic using the software Epi Info 6.04. Percentage of global agreement was also performed and reported as a percentage with its respective 95% confidence interval.

For the purposes of this work, an agreement of 80% or greater is considered acceptable, since these are diagnostic performance studies.

## Results classification and analysis

The mass spectrometry results were compared with the identifications obtained by the gold-standard method and were classified as follows: correct species identification, correct genus identification, correct group or complex identification, NI. We recorded the cases in which the identification failure was due to the absence of a species protein profile in the DB and when the limitation in the identification had already been described by the manufacturers. Those cases in which it was not possible to obtain reproducible spectra (No Peaks result) in spite of testing different extraction methods, were also detailed.

When faced with a discrepancy, it was decided to repeat the analysis in both systems in order to eliminate the possibility of contamination or operator error.

Out of a total of 265 isolates, representing 143 species: Microflex LT correctly identified 83% (220/265) at the genus / complex level; 64% (170/265) at the species level, while only 17% (45/265) were NI.

**Fig 1:**
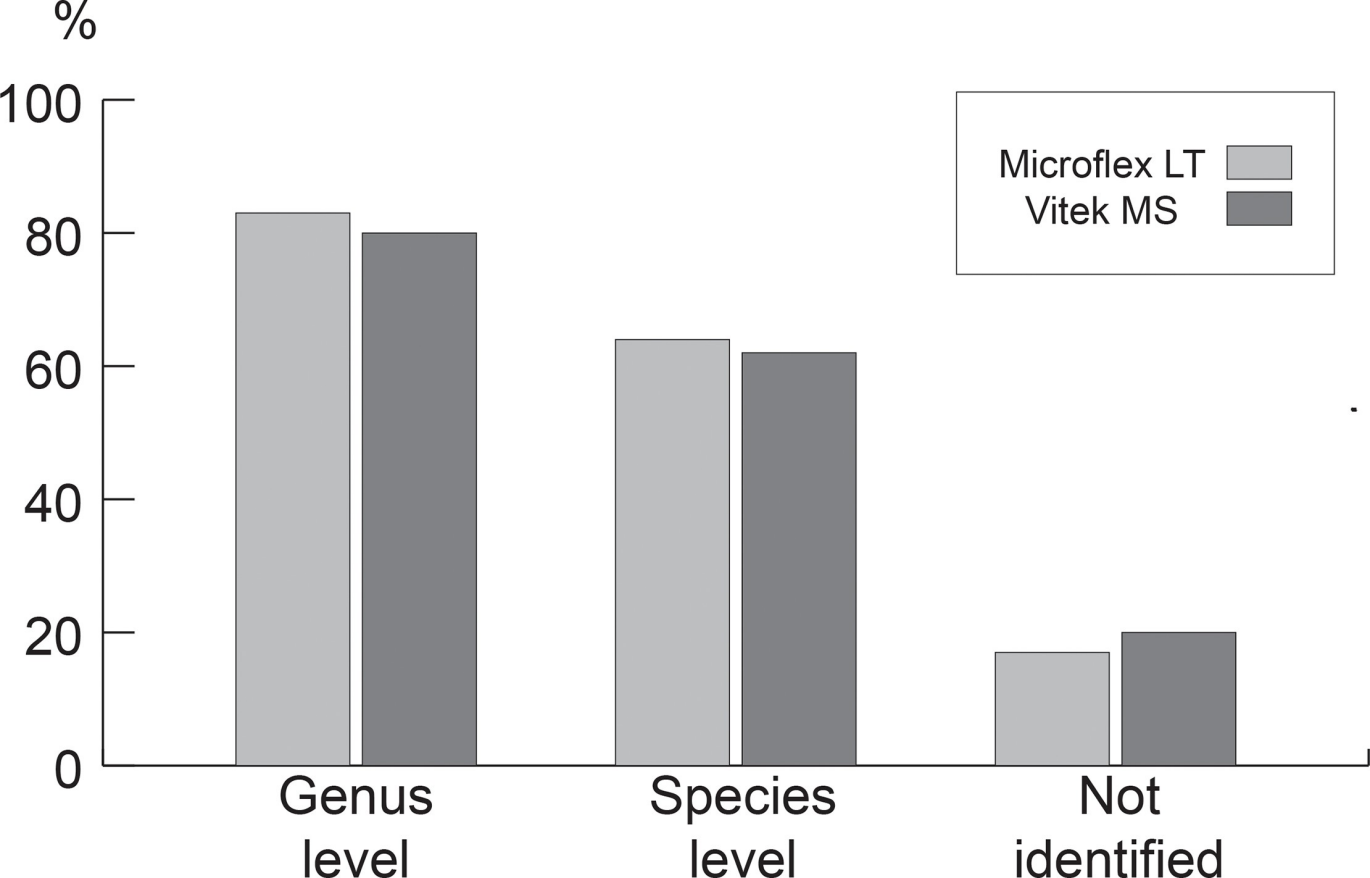
Performance of both platforms on the total of microorganisms tested. Percentage of identification at the species level, genus and not identified by both instruments.

For the Vitek MS platform, 80% (212/265) of the isolates were identified at the genus / complex level, 62% (164/265) at the species level and 20% (53/265) were not identified (Fig 1).

The concordance calculated, was very good for identifications at genus and species level; only minor percentages of agreement were observed between Gram Positive Cocci at the species level and between Gram Negative Bacilli at the genus level, this information can be found in Supplementary S2 Table (S2 Table).

### Gram-Positive Bacilli

The identification results in Vitek MS and Microflex LT, compared with the reference identification can be found in **Table 1**.

**Table 1:** Identification results in Vitek MS and Microflex LT for Gram-Positive Bacillus species.

REFERENCE ID ^a^		VITEK MS				MICROFLEX LT			
		COMPLEX ID	GENUS ID	SPECIES ID	NI	COMPLEX ID	GENUS ID	SPECIES ID	NI
Genus	Species (n)								
**Bacillaceae family**									
*Bacillus*	*B*. *amyloliquefaciens* (2)				2^c^				2^b^^,^^c^
	*B*. *cereusgroup* (2)	2				2			
	*B*. *licheniformis* (3)			2	1^b^			1	2^b^
	*B*. *pumilus*		1				1		
**Brevibacteriaceae family**									
*Brevibacterium*	*B*. *casei*		1					1	
**Lactobacillaceae family**									
*Lactobacillus*	*L*. *fermentum* (2)			2				1	1^b^
	*L*. *delbrueckii*			1				1	
	*L*. *gasseri*		1					1	
	*L*. *paracasei*		1					1	
	*L*. *rhamnosus*		1					1	
*Pediococcus*	*P*. *pentosaceus*			1				1	
**TOTAL**		**2**	**11**	**6**	**3**	**2**	**9**	**8**	**5**

**a.** Identified by sequencing the 16S r*RNA* gene as a gold standard.

**b.** NI: Not identified despite being present in the commercial Database.

**c.** NI: Not Identified due to faults in the generation of the spectrum (no peaks). COMPLEX ID: correct identification at the group / complex level; GENUS ID: correct identification at genus level; SPECIES ID: correct identification at species level; NI: no identification.

Vitek MS identified 81% (13/16) of the isolates at the genus level, 38% (6/16) at species level and 19% (3/16) were NI. Microflex LT reached 69% (11/16) of correct identifications at the genus level, 50% (8/16) at species level and 31% (5/16) NI.

It was not possible to achieve discrimination between the species from the *Bacillus cereus* group (including the species: *B*. *cereus*, *B*.*thuringiensis*, *B*. *mycoides*, *B*. *pseudomycoides*, *B*. *cytotoxicus*, *B*. *weihenstephanensis y B*. *toyonensis)* and the *Bacillus altitudinis–B*. *pumilus* group. These limitations have been described by the manufacturers, because such species share a significant degree of genetic resemblance, therefore, supplementary tests and taxonomic resolution are required for their differentiation **[36].**

Additionally, none of the two systems achieved sufficient reproducible spectra of *Bacillus amyloliquefaciens* despite having tested different extraction methods, which could be due to the cultural characteristics of the species. Microflex LT correctly identified most species of the *Lactobacillus* sp., while Vitek MS identified them as group: *Lactobacillus acidophilus/Lactobacillus gasseri* y *Lactobacillus casei/Lactobacillus paracasei/Lactobacillus rhamnosus*.

### Coryneiform bacteria

Of the total of coryneiform (n = 45) that were tested, Microflex LT identified 87% (39/45) at the genus level, 60% (27/45) at species level and 13% (6/45) were NI. While Vitek MS reached 78% (35/45) of identifications at the genus level, 62% (28/45) at species level and 22% (10/45) were NI **(Table 2).**

**Table 2:** Identification results in Vitek MS and Microflex LT for coryneiform species.

REFERENCE ID^a^		VITEK MS				MICROFLEX LT			
		COMPLEX ID	GENUS ID	SPECIES ID	NI	COMPLEX ID	GENERA ID	SPECIES ID	NI
Genus	Species (n)								
**Actinomycetaceae Family**									
*Actinotignum*	*A*. *schaalii*			1			1		
*Actinomyces*	*A*. *naeslundii* (2)		1	1			1		1^b^
	*A*. *odontolyticus*			1					1^b^
	*A*. *radingae* (2)			2				2	
	*A*. *turicensis*			1				1	
*Trueperella*	*T*. *bernardiae* (2)			2			1	1	
**Cellulomonadaceae Family**									
*Cellulomonas*	*Cellulomonas hominis*				1				1
**Promicromonosporaceae Family**									
*Cellulosimicrobium*	*C*. *cellulans*			1				1	
**Corynebacteriaceae Family**									
*Corynebacterium*	*C*. *amycolatum* (3)		3					2	1^b^
	*C*. *aurimucosum*				1^b^			1	
	*C*. *bovis*				1^b^			1	
	*C*. *diphteriae* (4)			3	1^b^		2	2	
	*C*. *jeikeium*			1				1	
	*C*. *kroppenstedtii* (2)			1	1^b^		1	1	
	*C*. *mucifaciens* (2)			1	1^b^		2		
	*C*. *propinquum*				1^b^		1		
	*C*. *striatum* (2)			1	1^b^		1	1	
	*C*. *tuberculostearicum*			1				1	
	*C*. *ulcerans* (2)		1	1			1		1^b^
	*C*. *urealyticum* (2)			1	1^b^^,^^c^			2	
	*C*. *xerosis* (2)		2					1	1^b^
*Turicella*	*T*. *otitidis* (2)			2				2	
**Dermabacteraceae Family**									
*Dermabacter*	*D*. *hominis*				1^b^			1	
**Erysipelotrichaceae Family**									
*Erysipelothrix*	*E*. *rhusiopathiae*			1				1	
**Bifidobacteriaceae Family**									
*Gardnerella*	*G*. *vaginalis* (2)			2				2	
**Listeriaceae Family**									
*Listeria*	*L*. *monocytogenes* (4)			4			1	3	
**TOTAL**			**35**	**28**	**10**		**39**	**27**	**6**

**a.** Identified by sequencing the 16S rRNA gene as a gold standard and *rpo*B gene.

**b.** NI: Not identified despite being in the commercial Database.

**c.** NI: Not Identified due to faults in the generation of the spectrum (no peaks).

Vitek MS could not differentiate *Corynebacterium amycolatum*—*C*. *striatum—C*. *xerosis*; Microflex LT, on the other hand, could not differentiate *Corynebacterium striatum—C*. *amycolatum; Corynebacterium mucifaciens—C*. *ureicelerivorans and Corynebacterium propinquum—C*. *pseudodiphteriticum* (limitations described by both manufacturers).

A *Cellulomonas hominis* isolate was not identified since it was not represented in the commercial DB of both platforms. The species that cause diseases in humans are absent in the commercial Database so they can get low score values; the definitive identification of these species is often carried out through molecular biology.

*Dermabacter hominis* was misidentified as *Corynebacterium durum* despite being present in the IVD 3.0 DB; whereas it was correctly identified with the Bruker system.

Of the coryneiform that did not yield reproducible results in the IVD DB, the identification did not improve when they were compared with the reference spectra of the RUO DB.

The majority of the *Corynebacteria / Actinomyces* that were not identified, are unusual in clinical samples, so they are absent or poorly represented in commercial libraries **[37].**

All *Listeria monocytogenes* tested were identified at species level. However, since other species were not included, it was not possible to establish the real accuracy of this technique for the identification of Listeria.

### Gram-Positive Cocci

The Vitek MS platform correctly identified 79% (26/33) of the isolates at the genus level, 67% (22/33) at species level and 21% (7/33) were NI, while Microflex LT reported 76% (25/33) at genus level, 52% (17/33) at species level and 24% (8/33) were NI.

Among the Gram-Positive Catalase-Positive Cocci; both platforms showed a good performance. A *Rothia dentocariosa* isolate was identified at genus level *(Rothia aeria)* in both systems, and *Kocuria rosea* was identified as *Kocuria rosea/polaris* in Microflex LT.

No identification was obtained with a single isolation of *Micrococcus luteus*, despite being present in both commercial libraries. **(Table 3).**

**Table 3:** Identification results in Vitek MS and Microflex LT for Gram-Positive Cocci species.

REFERENCE ID ^a^		VITEK MS				MICROFLEX LT			
		COMPLEX ID	GENUS ID	SPECIES ID	NI	COMPLEX ID	GENUS ID	SPECIES ID	NI
Genus	Species (n)								
**Micrococcaceae Family**									
*Arthrobacter*	*Arthrobactersp*				1^b^			1	
*Kocuria*	*K*. *kristinae*			1				1	
	*K*. *rosea (2)*			2			2		
*Micrococcus*	*M*. *luteus*				1^b^				1^b^
*Rothia*	*R*. *dentocariosa*		1				1		
	*R*. *mucilaginosa (2)*			2				2	
**Aerococcaceae Family**									
*Dolosicoccus*	*D*. *paucivorans*				1				1
*Facklamia*	*F*. *soureckii*				1				1
	*F*. *hominis*			1				1	
*Ignavigranum*	*I*. *ruoffiae*				1				1
**Enterococcaceae Family**									
*Enterococcus*	*E*. *casseliflavus*			1				1	
	*E*. *durans*			1				1	
*Vagococcus*	*V*. *fessus*				1		1		
**Carnobacteriaceae Family**									
*Granulicatella*	*G*. *elegans*				1^b^				1^b^
	*G*. *adiacens*			1					1
**Peptoniphilaceae Family**									
*Helcococcus*	*H*. *kunzii* (3)			3				3	
**Leuconostocaceae Family**									
*Leuconostoc*	*L*. *mesenteroides*			1				1	
*Weissella*	*W*. *confusa*			1					1^b^
**Streptococcaceae Family**									
*Lactococcus*	*L*. *garvieae*			1				1	
	*L*. *lactis*			1				1	
*Streptococcus*	*S*. *gallolyticus* ssp *pasteurianus*			1				1	
	*S*. *lutetiensis* (2)			2		1		1	
	*S*. *mitis*(2)		2			1	1		
	*S*. *mutans*			1				1	
	*S*. *pneumoniae*			1					1^b^
	*S*. *pseudopneumoniae*		1				1		
	*S*. *suis*			1				1	
**TOTAL**			**26**	**22**	**7**	**2**	**23**	**17**	**8**

a. Identified by sequencing the 16S *ARN*r gene as a gold standard

b. NI: Non-identified despite being in the commercial Database.

Among the Gram-Positive Catalase-Negative Cocci, a better diagnostic efficiency was observed at species and group / complex level in the Vitek MS equipment, both for more-common as for the less-frequent microorganisms **(Table 3)**. This meant higher confidence levels, and it was not necessary to use RUO DB.

In the same way, mass spectrometry was very useful in the cases of species like *Granulicatella* sp., *Lactococcus* sp. y *Vagococcus* sp., that have a slow and cumbersome identification. **[38].**

One of Microflex LT known difficulties is the limitation in the identification of the closely-related species *Streptococcus mitis / oralis / pneumoniae*, as it has been described by other authors **[7, 39, 40].**

Vitek MS showed a great performance in this group of microorganisms without the need of being analyzed by the second software available. Among the few species that it was not able to identify we can find *Granulicatella elegans*.

The non-identified microorganisms were similar in both platforms and in most cases, they were not included in the commercial DB since they belong to rare species or non-frequent ones, therefore their clinical significance is under discussion.

### Anaerobes

Both platforms showed an excellent performance, with 85% (34/40) of correct identifications at the genus level, 83% (33/40) at the species level, 15% (6/40) NI); in most cases, yielding reliable results in a matter of minutes that would otherwise take much more time and money for the laboratory routine. **(Table 4)**

**Table 4:** Identification results in Vitek MS and Microflex LT for anaerobes species.

REFERENCE ID ^a^		VITEK MS				MICROFLEX LT			
		COMPLEX ID	GENUS ID	SPECIES ID	NI	COMPLEX ID	GENUS ID	SPECIES ID	NI
Genus	Species (n)								
**Bacteroidaceae Family**									
*Bacteroides*	*B*. *caccae*		1				1		
	*B*. *fragilis*			1				1	
**Clostridiaceae Family**									
*Clostridium*	*C*. *difficile* (14)			14				14	
	*C*. *perfringens* (11)			11				11	
	*C*. *septicum* (2)			2				2	
	*C*. *sporogenes*			1				1	
	*C*. *tertium* (2)			2				2	
**Peptostreptococcaceae Family**									
*Paeniclostridium*	*P*. *sordellii*				1^b^			1	
**Eggerthellaceae Family**									
*Eggerthella*	*Eggerthella lenta* (2)				2^b^				2^b^
**Porphyromonadaceae Family**									
*Porphyromonas*	*Porphyromonas* sp				1^b^				1^b^^,^^c^
**Propionibacteriaceae Family**									
*Pseudopropionibacterium*	*P*. *propionicum* (2)				2^b^				2^b^
*Cutibacterium*	*C*. *acnes*			1				1	
	*C*. *granulosum*			1					1^c^
**TOTAL**			**34**	**33**	**6**		**34**	**33**	**6**

**a.** Identified by sequencing the 16S *ARN*r gene as a gold standard

**b.** NI: Non-identified despite being in the commercial Database

**c.** NI: Non-identified due to faults in the generation of the spectrum (no peaks)

None of the systems was able to get the identification for *Pseudopropionibacterium propionicum* and neither of them achieved sufficient quality spectra for *Porphyromonas* sp. It should be noted that both platforms identified the *Bacteroides* species, both the *Bacteroides fragilis* and the *Bacteroides caccae* group, the anaerobic pathogens with higher rates of antimicrobial resistance. Based on the experience as a National Reference Laboratory and in accordance with the proposal by Legarraga et al., we consider necessary the improvement of the commercial DBs in order to increase the score values yielded for this group of microorganisms **[17].** However, when the protein profiles are included in the Databases, the identification by mass spectrometry is notoriously better than the conventional biochemical identification.

### Gram-Negative Bacilli

Most isolates were correctly identified by both systems at species or group / complex level, cases in which the sequencing of specific genes is usually necessary for the complete differentiation **(Table 5).** The Bruker platform correctly identified 88% (68/77) of the gram-negative bacilli at the genus level, 57% (44/77) at species level, and 12% (9/77) were non-identified; while the Vitek MS device yielded 73% (56/77) identifications at genus level, 48% (37/77) at species level and 27% (21/77) NI.

**Table 5:** Identification results in Vitek MS and Microflex LT for the Gram-Negative Bacilli clinically relevants.

REFERENCE ID^a^		VITEK MS				MICROFLEX LT			
		COMPLEX ID	GENUS ID	SPECIES ID	NI	COMPLEX ID	GENUS ID	SPECIES ID	NI
Genus	Species (n)								
**Moraxellaceae Family**									
*Actinobacillus*	*A*.*lignieresii*		1				1		
	*A*. *ureae* (2)			2				2	
*Moraxella*	*M*.*bovis*	* *	1	* *	* *	* *	* *	1	* *
**Alcaligenaceae Family**									
*Achromobacter*	*A*.*spanius*		1				1		
	*A*.*xylosoxidans* (4)		4				3		1^c^
*Advenella*	*A*.*incenata*				1			1	
*Alcaligenes*	*A*.*denitrificans*		1				1		
*Bordetella*	*B*.*parapertussis*			1				1	
**Alteromonadaceae Family**									
*Alishewanella*	*A*.*fetalis*				1				1^b^
**Flavobacteriaceae Family**									
*Bergeyella*	*B*.*zoohelcum*			1				1	
*Chryseobacterium*	*C*.*bovis*	* *	* *		1		1		
	*C*.*gleum* (3)	* *	* *	2	1^b^		2		1^b^
	*C*.*indologenes* (2)	* *	* *	2			1	1	
*Elizabethkingia*	*Elizabethkingia*sp (2)		2				2		
	*E*.*meningoseptica* (2)			2				2	
**Burkholderiaceae Family**									
*Burkholderia*	*B*.*cepacia* (3)^d^		2	1			3		
	*B*.*cenocepacia* (2)^d^		2				2		
	*B*.*contaminans* (11)^d^			11				11*	
	*B*.*multivorans* (3)^d^			3				3	
	*B*.*vietnamensis* (3)^d^		1	2				3	
	*B*.*seminalis*(3)^d^				3		2		1^c^
*Cupriavidus*	*C*.*metallidurans*		1					1	
*Pandoraea*	*P*.*sputorum*				1^b^				1^c^
*Ralstonia*	*R*. *insidiosa*			1				1	
	*R*.*pickettii* (2)			2				2	
**Chromobacteriaceae Family**									
*Chromobacterium*	*C*.*violaceum* (2)			2				1	1^c^
**Comamonadaceae Family**									
*Comamonas*	*C*.*kerstersii* (2)		1		1			2	
*Delftia*	*D*.*acidovorans*				1^b^			1	
	*D*.*tsuruhatensis*		1				1		
*Ramlibacter*	*R*.*henchirensis*				1				1
**Gammaproteobacteria Family**									
*Ignatzschineria*	*I*. *indica*				1			1	
**Rhodospirillaceae Family**									
*Inquilinus*	*I*.*limosus*			1				1	
**Brucellaceae Family**									
*Ochrobactrum*	*O*.grupo *anthrophi*			1				1	
**Rhodobacteraceae Family**									
*Pannonibacter*	*P*.*phragmitetus* (2)				2			2	
**Pseudomonadaceae Family**									
*Pseudomonas*	*P*.*chlororaphis*			1				1	
	*P*. *mendocina*		1				1		
	*P*.*mosselii*				1^b^		1		
	*P*.*oleovorans*				1^b^		1		
	*P*.*oryzihabitans* (2)			2				2	
	*P*.*otitidis*				1^b^			1	
**Acetobacteraceae Family**									
*Roseomonas*	*R*.genomoespecie 5 (2)				2		1		1
**Sphingobacteriaceae Family**									
*Sphingobacterium*	*S*.*lactis*				1				1
**Xanthomonadaceae Family**									
*Wohlfartimonas*	*W*.*chitiniclastica*				1			1	
**TOTAL**			**56**	**37**	**21**		**68**	**44**	**9**

**a.** Identified by sequencing the 16S ARNr gene as a gold standard

**b.** NI: Non-identified despite being in the commercial Database

**c.** NI: Non-identified due to faults in the generation of the spectrum (no peaks)

**d.** Characterized by the sequencing of gene *rec*A

* Identified with the supplementary RENAEM V2.0 database

The DB Biotyper 3.1 turned out to be more complete from the perspective of the GNB diversity, achieving a greater number of correct identifications at genus and species level. On the other hand, when a species is not represented in the commercial DB, the Microflex LT system tends to get results at genus level, while Vitek MS does not show identification.

From our experience, the Vitek MS platform turned out to be more sensitive to the quantity of the microorganism deposited in the sample preparation, however the readquisition of the spectra corresponding to samples that did not show a result in the first reading; improve the identification of several isolates such as of *Burkholderia cepacia* and *B*. *cenocepacia*. When comparing the profiles that kept showing NI with the ones from the Saramis (RUO) DB, improvements were obtained in the identification at the genus level for the *Pseudomonas otitidis* and *Chryseobacterium gleum* species.

The *Pseudomonas mendocina* species were identified at genus level by both platforms, Vitek MS showed *Pseudomonas oleovorans* as a result and Microflex LT, *Pseudomonas mendocina–P*. *alcaliphila*.

Both platforms identified at genus level the closely-related species *Achromobacter xylosoxidans—Alcaligenes denitrificans and Actinobacillus lignieresii—A*. *pleuropneumoniae*; these limitations were already described by the manufacturers, since identification at the species level is not achieved by common techniques.

*Cupriavidus metallidurans* was identified at genus level by Vitek MS, which showed *Cupriavidus pauculus;* whereas Microflex LT yielded a correct identification at species level. In several previous publications, the limitations of Vitek MS among Non-Fermenting Bacilli are reviewed, yielding misidentifications, such is the case of *Comamonas sp*., which can result in *Delftia acidovorans* **[37, 41, 42].**

*Burkholderia cepacia* complex. There are no reference profiles for *Burkholderia seminalis* species in the IVD Database, which usually yields, with high levels of confidence, identification with other species of the genus. But correctly identify *Burkholderia multivorans*, *B*. *vietnamensis* y *B*.*contaminans* species, being this one the most prevalent in the fibrocystic population in Argentina. On its behalf, the Bruker platform does not have representative spectra for this important species. Presently, the *rec*A gene sequencing is the gold-standard method, but it is costly, laborious and is not likely to be applied in a clinical laboratory routine. It was possible to optimize the MALDI TOF MS methodology for the identification of B. *contaminans* by creating a database of local isolates completely characterized spectra to be used in addition with the Biotyper 3.1 commercial database. The generation and incorporation of the reference protein profiles were carried out as described by Cipolla et. al.**[43].** This new generated database was denominated RENAEM V2.0 and was transferred and validated by external reference centers. The additional database allowed a reliable identification for all of the *B*. *contaminans* isolates, and was comparable to the gene *rec*A sequencing method, but with a substantial reduction of time, supplies and hard work in order to get a result. This constitutes the first database of protein spectra created in Argentina for the improvement of the bacterial identification of species from the *Burkholderia cepacia* complex by the MALDI TOF MS technology.

The *Roseomonas* isolates that were tested, showed identification problems, probably due to the cultural characteristics (mucous, pigmented) of the species.

### Actinomycetales

The results for both platforms, regarding the reference identification (16S *ARN*r gene and *sec*A gene) are shown in **Table 6**. Vitek MS correctly identified 69% (24/35) of the isolates at the genus level, 46% (16/35) at the species level and 31% (11/35) were NI; while Microflex LT managed to identify 74% (26/35) of the actinomycetales tested at the gender level, 49% (17/35) at the species level and 26% (9/35) NI. The majority of the NI occurred due to the difficulty in creating quality spectra that could be compared with the reference profiles, and this is essentially due to the complex chemical composition of its cell wall. The objective was to assess the extraction methods recommended and we concluded that there are not necessary complex procedures if the conditions are optimized, by carrying out simple preliminary steps of extraction on fresh colonies. In general, the results showed that MALDITOF MS is effective for the identification of the Actinomycetales species, but the supplementation with customized spectra can substantially improve the performance of the current commercial DBs.

**Table 6:** Identification results in Vitek MS and Microflex LT for the most frequent clinically-relevant Actinomycetal species.

REFERENCE ID^a^		VITEK MS				MICROFLEX LT			
		COMPLEX ID	GENUS ID	SPECIES ID	NI	COMPLEX ID	GENUS ID	SPECIES ID	NI
Genus	Species (n)^a^								
**Gordoniaceae Family**									
*Gordonia*	*G*. *bronchialis*			1					1^c^
	*G*. *terrae*				1^b^				1^b^
**Nocardiaceae Family**									
*Nocardia*	*N*. *abscessus* (4)		1	1	2^b^		1	2	1^b^
	*N*. *brasiliensis* (3)			3				2	1^b^
	*N*. *cyriacirgeorgica* (6)			6				5	1^b^
	*Complejo N*. *exalbida–N*. *arthritidis–N*. *gamkensis* (9)		6		3^b^		7		2^c^
	*N*. *farcinica* (4)			4				4	
	*N*. *wallacei*			1			1		
*Rhodococcus*	*R*. *hoagii* (2)		1		1^b^				2^b^^,^^c^
**Tsukamurellaceae Family**									
*Tsukamurella*	*T*. *paurometabola* (4)				4^c^			4	
**TOTAL**			**24**	**16**	**11**		**26**	**17**	**9**

**a.** Identified by sequencing the 16S ARNr gene as a gold standard and gene *sec*A.

**b.** NI: Non-identified despite being in the commercial Database

**c.** NI: Non-identified due to faults in the generation of the spectrum (no peaks)

For Microflex LT, the best results were obtained with the direct method and with the EFAE Bruker extraction technique.

In the Vitek MS system, the extraction kit supplied by the manufacturer was simple and easy-to-use, however, it was not possible to obtain enough reproducible spectra in any case, except for one *Nocardia wallacei* isolate. The rest of the extracts obtained, none of them yielded results in the IVD DB, improving the identification in only three isolates when challenged in RUO DB; these belonged to *Nocardia farcinica*, *Gordonia bronchialis* y *Rhodococcus* sp.

On the other hand, the supernatants obtained with the extraction kit for Actinomycetales of Vitek MS were processed in the Bruker Microflex LT system, obtaining identification at least at genus level in 12/18 isolates; in 5 samples no peaks were obtained and identification was not achieved in one well.

Likewise, and in order to improve the identification in Vitek MS; the same isolates were tested using the ***in-house*** protocol of disruption in the well with formic acid. The results were significantly better when processing the samples with this simple and inexpensive procedure. In conclusion, Vitek MS yielded reproducible results when processing the samples using the direct method and the disruption method in the well. Both platforms correctly identified *Nocardia brasiliensis*, *Nocardia farcinica*, *Nocardia cyriacigeorgica* at species level; these correspond to species that are generally multi-resistant to antimicrobials. The rest of the *Nocardia* spp. were identified to the genus level; for the species from the *Nocardia exalbida–N*. *arthritidis–N*. *gamkensis* complex, Vitek MS showed *Nocardia beijingensis* as a result; while Microflex MT showed them as *Nocardia abscessus–N*. *araoensis–N*. *pneumoniae*.

### HACEK

Fastidious microorganisms were correctly identified with high confidence levels for both platforms, with identifications at genus and species level close to 100%; which reveals the benefit of mass spectrometry over conventional methods in inert strains and of-difficult-development **(Table 7).**

**Table 7:** Identification results in Vitek MS and Microflex LT compared with the reference identification for fastidious microorganisms species.

REFERENCE ID^a^		VITEK MS				MICROFLEX LT			
		COMPLEX ID	GENUS ID	SPECIES ID	NI	COMPLEX ID	GENUS ID	SPECIES ID	NI
Genus	Species (n)								
**Pasteurellaceae Family**									
*Aggregatibacter*	*A*.*actinomycetemcomitans*	* *		1				1	
	*A*. *aphrophilus* (2)	* *		2				2	
*Haemophilus*	*H*.*parainfluenzae* (3)	* *	1	2			1	2	
*Histophilius*	*H*. *somni* (2)	* *		2				2	
*Mannheimia*	*M*. *haemolytica*	* *		1				1	
*Pasteurella*	*P*. *canis* (2)	* *		2				2	
	*P*.*multocida*	* *		1				1	
**Flavobacteriaceae Family**									
*Capnocytophaga*	*C*.*sputigena* (3)			3				3	
**Cardiobacteriaceae Family**									
*Cardiobacterium*	*C*. *hominis*			1				1	
**Neisseriaceae Family**									
*Eikenella*	*E*.*corrodens*			1				1	
*Kingella*	*K*.*kingae*			1				1	
*Neisseria*	*N*.*bacilliformis*				1			1	
**TOTAL**			**18**	**17**	**1**		**19**	**18**	

**a.** Identified by sequencing the 16S ARNr gene as a gold standard and gene *sec*A.

### Comparison of technical aspects

In a similar way to the evaluation in Chile by Porte et al. **[19],** the technical differences between both platforms were evaluated and the results are summarized in **Table 8.**

**Table 8:** Comparison of technical and practical aspects between both platforms.

MICROFLEX LT	VITEK MS
Tabletop equipment	Floor equipment
Reusable plate with 96 wells	Disposable plates with three acquisition groups of 16 wells (48 wells per plate).
HCCA, lyophilized. Volume: 250ul	HCCA, ready to use. Volume: 500ul
BTS, Bruker Test Standard Calibration	*E*. *coli* ATCC 8739 standard strain calibration
Optional: Extraction with Formic Acid	Optional: Extraction with Formic Acid
*Shigella / E coli* Limitations	*Shigella / E coli* Limitations
More sensitive to the amount of microorganism in the well	Less sensitive to the amount of microorganism in the well
More complete DB (more genus and species)	Less complete DB
Less incorrect identifications when absent from the DB	Higher number of incorrect identifications when absent from the DB
Results expressed in score values	Results expressed in identification %
Easy-to-use software	Easy-to-use MYLA software connected to the other automated laboratory systems. Saramis Premium is less friendly for routine application and complex review of results
Shows the result in less time; faster reading and yields result per well read	Takes longer to show results, longer reading time and result is only available at the end of the run in an acquisition group

Microflex LT is a tabletop instrument so it requires less laboratory space and provides greater comfort to load the plate into the equipment, while Vitek MS is floor equipment that occupies greater space and the operator has to bend to introduce the samples that will be processed. Sample preparation by the direct method was similar, but for a careful manipulation of the *Escherichia coli* ATCC 8739 strain, which was used as a calibrator and internal quality control in Vitek MS. However, the availability of disposable plates and reagents ready to be used shortens time and reduces the probability of pre-analytical errors and contamination.

Vitek MS allows the simultaneous processing of four plates that can be seeded in different areas of the laboratory, which gives it greater analytical capacity.

Regarding speed, Microflex LT takes less time to reach the vacuum level and to process the samples, which may be due to the shorter length of the flight tube of the equipment; the analysis of the results can be done as the system processes each sample individually and allows the observing of the first ten score results for each microorganism, even when reliable identification was not obtained. In Vitek MS, the results appear per read quadrant, i.e. every 16 wells, and the system gives a single possible result.

Vitek MS yields a greater number of multiple results like complex/species that lead to fewer mistakes when it comes to closely related species. This grouping style favors the analysis from clinical samples, since distinguishing these strictly related species tends to be clinically irrelevant; however, it can cause certain limitations for research studies and the analysis of environmental, food and industrial samples **[37].**

Both systems differ in the composition of their Databases, the algorithms used for the identification and the tools. MALDI Biotyper 3.1 seemed more complete from the species diversity point of view. The classifications are reported in different scales, which is why they cannot be compared directly. In any case, both systems allow the user to add sets of spectra from local biological variants completely characterized, so they can build their own DB and therefore improve the performance of the identifications for specific taxonomic groups **[44].**

## Discussion

Since the introduction of mass spectrometry, routine microbiological diagnosis has reached a new era; it provides faster and more reliable results and reduces the need of performing complex techniques that occupy personnel and adds costs to the laboratory. This is why a great number of health institutions throughout the world have incorporated this new technology; however, an important aspect that should be considered is the existence of two systems available in the market, and what their performance with spectra of local biological variants and South America’s laboratory infrastructure is like.

To carry out the challenge, both platforms were set up in the National Reference Center INEI ANLIS “Dr. Carlos G. Malbrán” during a four-month period, where all samples were tested in parallel. In general, no significant differences were observed in the analytical performance of the DB of both systems for the identification of routine isolations. On its behalf, Vitek MS showed better efficiency in the identification of Gram-positive cocci; these results are similar to those published in previous studies **[39, 40].** Additionally, based on the bibliography and the experience of National Reference Laboratories in Argentina regarding Microflex LT, score values ≥ 1,70 could be accepted as a reliable result at species level for this group of microorganisms **[45]**.

Gram-negative bacilli were correctly identified at species or group/complex level by both systems, considering their shared limitations; such is the case of *Burkholderia cepacia* complex and *Achromobacter* species. There were slight differences in favor of the Bruker platform that could be attributed to a greater diversity of reference spectra in its DB and to the susceptibility of Vitek MS to the amount of sample at the time of processing.

In these cases where the complete identification is troublesome, even with molecular techniques, more research is required in order to detect whether said limitations were overcome with the increase of referent profiles of local variants completely characterized in the commercial Databases.

One of the main advantages of the MALDI TOF technology is the reliable identification of certain clinically-relevant microorganisms that are classified at the gender level by conventional methods and require molecular techniques or biochemical tests that take a long time; like the Bacteroides, some Corynebacteria and fastidious species **[14, 46–48]**. Likewise, anaerobic bacteria were identified with a high level of confidence and the few faults were due to the lack of representation in commercial libraries, so we believe it is necessary to have an update with a greater number of representative isolates for most-frequent clinically-relevant anaerobic species.

Among the bacteria from the *Nocardia* genus, the *N*. *farcinica* and *N*. *nova* species are the most frequently isolated, followed by *N*. *brasiliensis* and *N*. *cyriacigeorgica*.

Because the antimicrobial susceptibility and pathogenicity differ according to the species, from a clinical perspective, the results of bacterial identification must be reported quickly, similar to the minimum inhibitory concentration **[46]**. The application of MALDI TOF technology has greatly improved its identification, although there are limitations due to the chemical composition of the cell wall of these microorganisms; which directly affects the protein signal of the obtained spectrum and an insufficient representation in the commercial platforms; so, it is very important to choose an appropriate extraction method.

Regarding the Databases, the performance of the Saramis Premium (RUO) software was globally poorer. In conclusion, for routine procedures, the Bruker Biotyper and Vitek MS IVD systems are equally good in terms of analytical efficiency. It should be noted that the variations in electricity, temperature, and humidity equally affect the performance of both equipment. Other factors, like the price, the workflow and the laboratory activity will affect the choice of one system or the other **[39]**.

All the results of the experience have been transferred to the participants of the National Mass Spectrometry Network (http://www.anlis.gov.ar/renaem/), contributing to the equity of the health system in Argentina.

## Conclusions

Both MALDI TOF systems showed a high performance and strength, being faster, more accurate and more economic than conventional biochemical tests or molecular techniques.

Most of the isolates were correctly identified by both devices. For non-identified microorganisms, each case should be evaluated particularly, to assess whether it is possible to overcome such limitation with the increase of the number of reference profiles in the commercial Databases.

The analysis of the facilities and other technical aspects was generally positive and showed minor differences that depend, almost exclusively, on the infrastructure of each laboratory.

In general, MALDITOF MS represents a paradigm shift for clinical microbiology in both industrialized and non-industrialized countries.

Towards the near future, it is foreseen that this technology will be used for the detection of bacterial resistance determinants and pathogenicity factors, among others. Once the validations for these applications are achieved, MALDITOF MS will undoubtedly be the most-used methodology in all microbiological diagnostic laboratories.

## Supporting information

S1 Table Origin of the isolates that were used in the test. 265 isolates were used from clinical samples of human infections.
(DOCX)

S2 Table Percentage of global agreement (PGA), confidence interval and concordance (kappa value) obtained for Vitek MS-Microflex LT. We calculate the concordance for identifications at genus and species level between both systems.
(DOCX)
