# Supplementary material for: Utility of platforms Viteks MS and Microflex LT for the identification of complex clinical isolates that require molecular methods for their taxonomic classification
Source: PLoS One. 2019 Jul 3;14(7):e0218077. doi: 10.1371/journal.pone.0218077 (PMC6608940; doi:10.1371/journal.pone.0218077)
Supplement: S1 Table — 265 isolates were used from clinical samples of human infections. (DOCX) [file pone.0218077.s001.docx]

**Table S1**. Origin of the isolates that were used in the test.

| **Source of origin (n)** | | **Number of isolates** |
| --- | --- | --- |
| Sterile fluids (18) | Cerebrospinal fluid (CSF)  Bronchoalveolar lavage (BAL)  Pleural, peritoneal, articular fluid | 8  3  7 |
| Non-sterile fluids (22) | Urine  Fecal matter | 8  14 |
| Skin and soft tissues (60) | Wounds  Abscesses, lymph nodes, cellulitis  Heart valves | 13  45  2 |
| Blood (70) | Blood culture  Retroculture | 69  1 |
| Sputum |  | 53 |
| Unknown |  | 3 |
| Sample collection |  | 39 |
| **Total** |  | **265** |
