# Supplementary material for: Utility of platforms Viteks MS and Microflex LT for the identification of complex clinical isolates that require molecular methods for their taxonomic classification
Source: PLoS One. 2019 Jul 3;14(7):e0218077. doi: 10.1371/journal.pone.0218077 (PMC6608940; doi:10.1371/journal.pone.0218077)
Supplement: S2 Table — We calculate the concordance for identifications at genus and species level between both systems. (DOCX) [file pone.0218077.s002.docx]

**Table S2**. Percentage of global agreement (PGA), confidence interval and concordance (kappa value) obtained for VITEK MS-MICROFLEX LT.

| **Taxonomic Group** | **PGA Genus** | **95% CI** | **Concordance (kappa)** | **PGA Species** | **95% CI** | **Concordance (kappa)** |
| --- | --- | --- | --- | --- | --- | --- |
| Gram Negative Bacilli | 84,00 | (0.27-0.77) | Moderate (0.52) | 91,00 | (0.700.95) | Very good (0.82) |
| Gram Positive Cocci | 97,00 | (0.75-1.00) | Very good (0.91) | 85,00 | (0.45-0.94) | Good (0.69) |
| Anaerobes | 100,00 | 1.00 | Very good | 100,00 | 1.00 | Very good |
| Actinomycetales | 94,00 | (0.67-1.00) | Very good  (0.86) | 97,00 | (0.83-1.00 | Very good (0.94) |
| HACEK | 95,00 | na* | Very good | 95,00 | na* | Good |
| Gram Positive Bacilli | 97,00 | (0.76-1.00) | Very good (0.90) | 98,00 | (0.90-1.00) | Very good (0.97) |

*na=not apply. Kappa values significantly different from zero.
